# First person – Femke (Fen) van Rhijn-Brouwer

**DOI:** 10.1242/dmm.050861

**Published:** 2024-05-24

## Abstract

First Person is a series of interviews with the first authors of a selection of papers published in Disease Models & Mechanisms, helping researchers promote themselves alongside their papers. Fen van Rhijn-Brouwer is first author on ‘ Systematic review and meta-analysis of the effect of bone marrow-derived cell therapies on hind limb perfusion’, published in DMM. Fen is a PhD candidate in the lab of Marianne Verhaar at the University Medical Center Utrecht, The Netherlands, investigating how to use cell-based regenerative medicine strategies to treat vascular inflammation.


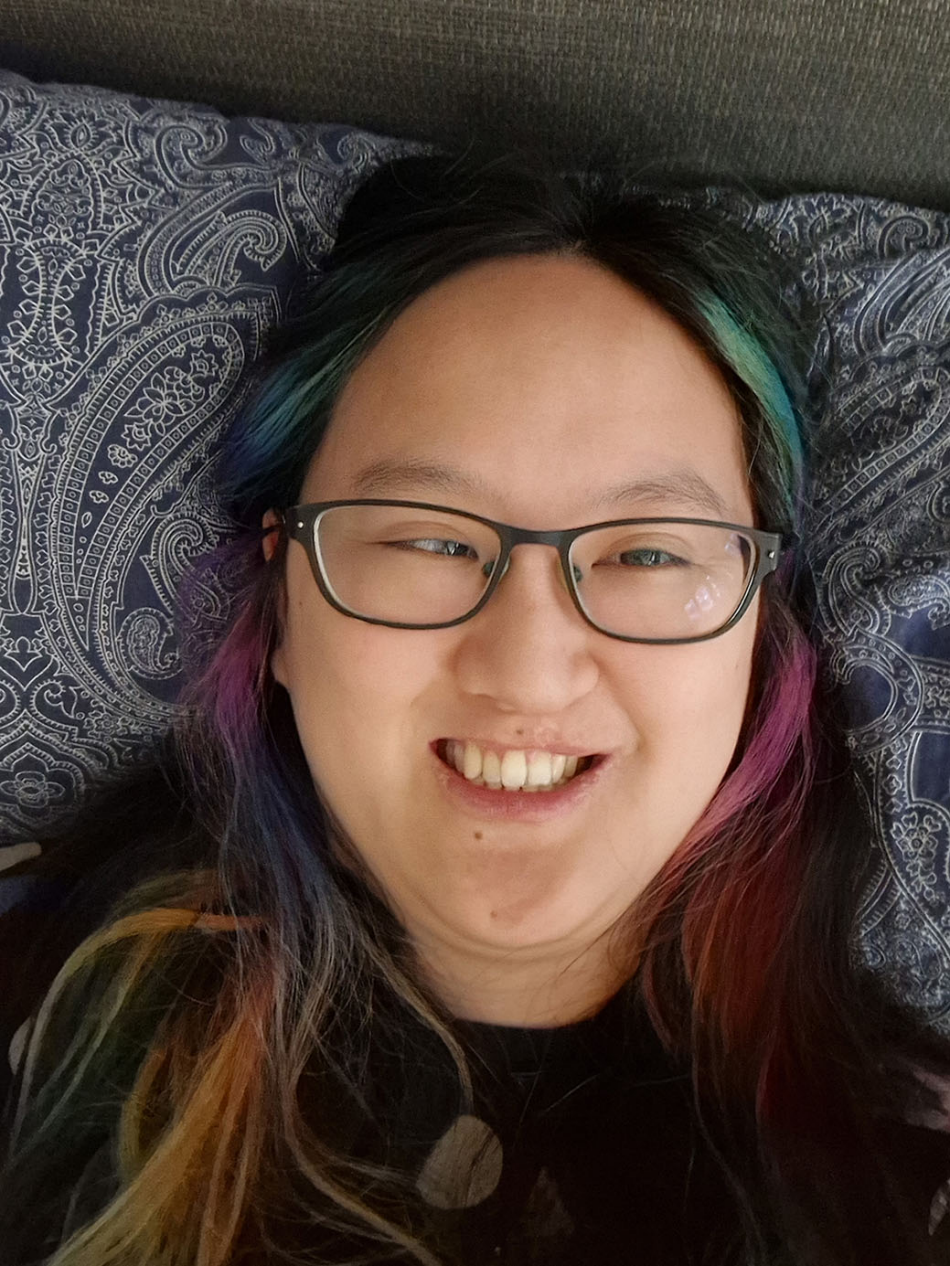


**Fen van Rhijn-Brouwer**

**Who or what inspired you to become a scientist?**

My sister has a severe mental disability and that inspired me to learn more about inflammation and the brain. I ended up in a different field, but that's where it all started, combing through research papers with my parents to figure out if we could do something to help her.

**What is the main question or challenge in disease biology you are addressing in this paper? How did you go about investigating your question or challenge?**

We conducted a systematic review and meta-analysis to gain more insight in the body of evidence for bone marrow cell-based therapies. This was prompted by the fact that human trials using bone marrow cell-based treatments for critical limb threatening ischemia were negative. We were looking for possibly overlooked factors, such as the donors, cell dose, administration route and recipient factors.

**How would you explain the main findings of your paper to non-scientific family and friends?**

We found that bone marrow cell-based treatments had a positive effect on the blood flow in animals that had been operated upon to simulate poor blood flow in the limb. However, this effect varied per study and we were unable to determine what caused this variation. Additionally, we found no effect of the type of cells that was used, donor characteristics, recipient characteristics or the administration route. When we evaluated the quality of the research, we found that many studies did not report important items that are needed to perform such an evaluation. This means that future animal studies looking into these treatments need to be carefully designed and planned.

The hind limb ischemia model comes with a lot of pain and discomfort for the animals. If it then doesn't yield reliable results, we might need to reconsider the model altogether.

**Is there a future for the animal model you looked into?**

I am asking myself this question because the hind limb ischemia (HLI) model comes with a lot of pain and discomfort for the animals. If it then doesn't yield reliable results, we might need to reconsider the model altogether.

In our data, we were unable to look at model-related factors such as the specific method used to induce HLI, so we don't know if these results are due to the model itself or other factors. Future studies will have to look into this.

Therefore, I feel it is too early to retire the model altogether. However, as we are all committed to use the fewest animals possible and subject them to the lightest possible discomfort, it is important to keep reflecting on our methods.

**Figure:**
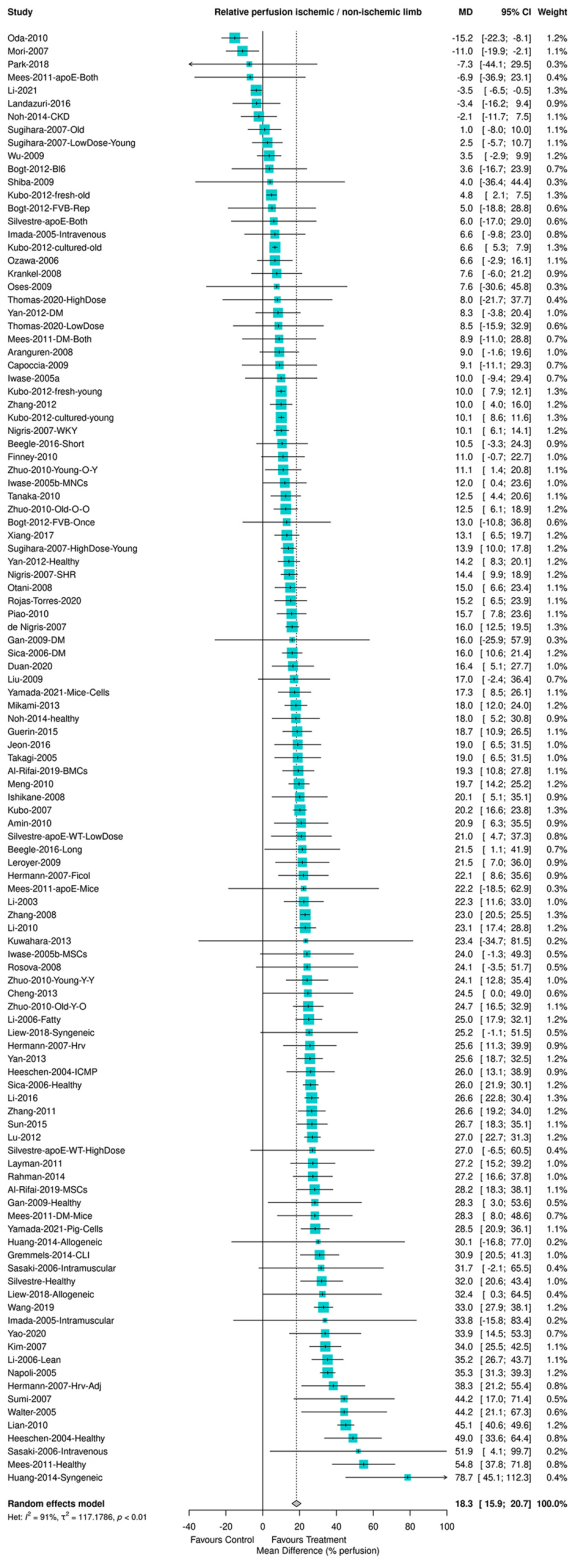
**Forest plot showing an increase in the relative maximum perfusion between the ischemic and the non-ischemic limb after treatment with BM-MNCs or BM-MSCs in animal models of hind limb ischemia.** Effects plotted as mean difference and pooled using a random-effects model. *n*=111 comparisons from 76 studies. BM-MNC, bone marrow-derived mononuclear cell; BM-MSC, bone marrow-derived mesenchymal stem cell.

**What are the potential implications of these results for disease biology and the possible impact on patients?**

As a researcher, it is important to be aware that your small animal study might one day inspire large-scale human clinical trials. Therefore, it is very important to report even the smallest details of your experiment so it can be compared to other studies.

Due to the variable effect of bone marrow cell treatment in animals, we cannot draw reliable conclusions for patients. The possible effect we found is most likely an overestimation.

**Why did you choose DMM for your paper?**

DMM is a journal that doesn't shy away from harder-to-interpret meta-analyses. Our team also likes the focus on animal models, that perfectly suited our paper.

**Given your current role, what challenges do you face and what changes could improve the professional lives of other scientists in this role?**

I am currently ill with long COVID. Recent publications show that I am far from the only physician scientist battling this disease. Fortunately, my research team has been very supportive. While we are waiting for better treatments, support at work is essential to still feel a part of the scientific community. This ranges from workplace accommodations and working remotely for those who can still work, to peer support so you feel less alone. Every email or WhatsApp message I get from my colleagues brightens my day.

As scientists, we are also role models for the public regarding vaccination and other preventative measures such as mask wearing and creating safe air spaces at work.

**What's next for you?**

Recovering from long COVID now has the highest priority. I hope to be able to return to my job as a rheumatology resident, and finishing the PhD thesis is also very high on the bucket list. I love working at the bedside as well as at the lab bench, my patients have inspired many research projects. The field of vascular inflammation is at a very exciting stage where many new connections between the immune system and the vascular system have been identified. I hope to contribute to effective treatments and diagnostics in the future.

**Tell us something interesting about yourself that wouldn't be on your CV**

I have written several novels and hope to some day publish one.
